# Long non-coding RNA MCM3AP-AS1 drives ovarian cancer progression via the microRNA-143-3p/TAK1 axis

**DOI:** 10.3892/or.2021.8129

**Published:** 2021-07-01

**Authors:** Jihong Wen, Shumei Han, Man Cui, Yanli Wang

Oncol Rep 44: 1375-1384, 2020; DOI: 10.3892/or.2020.7694

Following the publication of this article, the authors attention was drawn to the fact that Table I and Fig. 6 contained some errors: The former contained some incorrect data, whereas the latter contained some inappropriately selected tumor images.

Following a further investigation in the Editorial Office, it has come to light that there were other possible anomalies associated with the presentation of the tumor images, and also that parts of the figure may have been published previously. Taking everything into consideration, the Editor has decided that the article should be retracted from the publication due to a lack of confidence in the data presented in this article. The authors were asked for an explanation to account for these concerns, but the Editorial Office did not received any reply. The Editor regrets any inconvenience that the retraction of the paper will cause.

